# Therapœia

**Published:** 1826-04

**Authors:** 


### Therapœia.

Pharmaca nulla valent, nisi quæ sint commoda causis.

32. *Empyema**. In an able paper " on the Indications and Treatment of Empyema," Dr. Hastings of Worcester has related several interesting cases of empyema, and deduced from them a series of conclusions. We present to our readers the substance of the cases, together with a few observations.

We quite agree with Dr. H's introductory remark, " that we are too much disposed to abandon, without any attempt at relief, prolonged cases of disease in the cavity of the chest, often erroneously judging that so *much mischief* has been produced in the structure of the organs contained in this cavity, as to render the continuance of life for any long period, altogether impossible. There are few individuals, who have been much in the habit of watching the progress of such affections, who cannot bring to their recollections, cases of this nature, in which, *after the doom of the patient has been declared inevitable, the sanative powers of the constitution have been alone sufficient to bring the disease to a happy determination.*"

After alluding to the great advancement made in our knowledge of diseases of the chest, and the precision with which we are now enabled to distinguish them, he observes, in reference to the stethoscope, " to say, that such precision of diagnosis is either not desirable, or that it will not lead to any practical important result, has always appeared to my mind the reverse of rational, and as bordering on the feeling that is *opposed* to every attempt at improvement, which does not bring with it some *immediate* and *certain advantage*,—a feeling than which nothing can be more inimical to the progression of science."

For some learned notices respecting the signification of the term empyema, the sense in which it was employed by the ancient as well as modern writers, and other historical information, we must refer our readers to the paper itself:—The cases and conclusions deduced from them are all that our limits will permit us to notice.

Case 1. *Empyema, conjoined with Tuberculous Affection of the Lungs.* " In May 1822, I visited R. A., æt. 19, in consultation with Mr. Watson of Stourport. In October, 1821, he was attacked with hæmoptoë, which lasted one day. Previous to the attack his breath had been short, and he had been affected with severe cough for several

* Edinburgh Journal of Medical Science, No. 1, for Jan. 1826, pp. 17—36

weeks.—From the attack of hæmoptoë till March 1822, he had a cough, and lost flesh. It was at this time that Mr. Watson first saw him, who applied blisters to the chest, and paid attention to the hepatic secretion. The pulse was under 100; the cough not very troublesome, excepting at night. The expectorated matter was small in quantity but had every appearance of purulence.

"In the beginning of April, a seton was inserted in the integuments of the chest. He took liquor potassæ and decoction of sarsaparilla twice a day, and occasionally a dose of blue pill. He continued this plan with slight variation until the time that I saw him (May), without any amendment being perceptible. It was now observed that the pulsation of the heart could only be felt on the right side of the chest, and he could not lie on that side. The pulse was 120, and wiry: the emaciation very great.

"By *percussion* the *left* side of the chest *sounded more* than the *right*, although there was not less sound than usual on the right side. By measurement, no difference was detected in the magnitude of the two sides of the chest.

"By the *cylinder, scarcely any respiratory murmur* was heard on the *left* side of the chest. The action of the heart was feebly audible in its usual situation, but most distinctly on the right side. The *respiratory murmur* was *very distinct* on the *right* side: it was in fact puerile in some parts. For some space above the *right* mamma, *pectoriloquism* was perfect.

"The combined results of the examination by the stethoscope and percussion, shewed that there was a *large tuberculous cavity* in the *right* lung, and that scarcely any air entered the *left* lung. This, from the displacement of the heart, and the inability to lie on the right side, appeared to arise from a *collection of fluid* in the *left sac* of the pleura. Percussion also seemed to indicate the presence of *air* in that side of the chest."

Before proceeding, we would remark upon this diagnosis, that whilst the *stethoscope* unequivocally proved the existence of *tuberculous cavities* in the *right* lung by the *perfect pectoriloquism* "above the right mamma," and also "that *scarcely any air entered the left lung*" by there being "scarcely any respiratory murmur" audible "on the left side of the chest," the *increased sound* of the *left* side elicited by *percussion*, indicated that there was not "a collection of *fluid*" but of *air* "in the left sac of the pleura."

As the tuberculous disease in this case prevented the possibility of the evacuation of the fluid producing any other benefit than that of temporary relief, it was resolved "that the operation should not be performed unless considerable dyspnœa came on from the pressure of the fluid." On the 17th of June Dr. H. again saw him. "His breath had become much worse, and the cough and expectoration were very troublesome."—"As he was now very anxious that the operation should be performed, it was agreed upon, after another examination with the cylinder and percussion, which afforded *similar results* to those before stated. We also employed Hippocratic succussion, and thereby distinctly heard the fluctuation of a fluid in the cavity of the chest.

"An incision was made with a scalpel in the space between the sixth and seventh ribs, near their angles, and nearly two quarts of dark-coloured sero-purulent fluid, together with a large volume of highly offensive gas were discharged. He bore the operation well, and expressed relief from it. On the second day after the operation a silver tube was introduced into the chest, and about a pint of fetid, purulent matter was discharged. He was much more easy in his breathing than he had been before the operation; but the cough and expectoration continued as before.—On the 25th of June, much gross purulent fluid escaped by the side of the tube.—He expired on the morning of the 28th."

*On Dissection*, the *heart* was nearly in its natural position but more to the right side than usual. It was *fluid (flaccid)* and the parietes very thin. The *left lung* hepatized, and compressed so as not to occupy one fourth part of the left cavity, had its pleural investment covered by a thick layer of viscid yellow lymph, in appearance not unlike chamois leather. The costal pleura was much thickened and covered with a false membrane. There was an ulcer in the pulmonic pleura, through which, when the lung was pressed, a fluid was discharged into the cavity of the chest. In the *right lung* there were numerous tubercles: the superior lobe adhered firmly to the first rib, and contained several tuberculous excavations.

We have already pointed out in regard to the diagnosis, that the indications of auscultation and percussion, as related by Dr. H., were those of effusion of *air* and not of *fluid* into the left cavity of the chest, and notwithstanding that *similar results* are said to have been obtained on the day of the operation, we feel quite satisfied that the side was more than *naturally sonorous only* in the *superior* portion of the chest when the patient was in the erect position, and in the *anterior* when lying down. Four pints of fluid in the cavity of the chest would undoubtedly occasion a *dull* sound in the *dependant* parts. Some of our readers may recollect Dr. Jackson's similar case (in our Number for Oct. 1825, page 542) in which two quarts of serous fluid were evacuated by operation: in it the *sound* on percussion *above* the the fourth rib was *unnaturally clear and loud*, whilst *below* it was unnaturally *flat and dead*. In one of M. Louis's cases, related in the Archives Générales (Juillet 1824, pp. 321—324) the chest gave a *very clear sound* from the nipple to the clavicle, and a *dead sound* in the other parts:—four pints of pus were found on examining the body.

Before we dismiss this case, we may remark that in a great majority of the instances of pneumothorax the air is derived from the brônchia by means of a tuberculous excavation opening through the pulmonary pleura. This occurrence is generally marked by a rather suddenly increased difficulty of breathing, and there are mostly indications of pleurisy;—the air soon compresses the lung, and the inflamed pleura pours out a serous fluid. If the stream of air bubbles forced by the act of inspiration through the perforation of the pleura has to pass through the fluid, it gives rise to metallic tinkling, a phenomenon exactly similar to the sound occasioned by forcing a stream of air through a small quantity of water contained in a barrel. This phenomenon does not commonly long continue, for as the lung becomes more and more compressed, the parietes of the tuberculous excavation are pressed together, and the communication is thereby wholly obstructed, or is opened only by strong efforts of coughing or forcible inspirations.

*Case* 2. *Empyema from Chronic Pleurisy, terminating fatally.* W. Osborne, æt. 23, was brought to the Worcester Infirmary, Jan. 1st. 1825, in consequence of a large abscess which had a short time before appeared on the right side of the spine, immediately below the inferior angle of the scapula. His health did not seem to be much affected; but he had a slight cough. Four days after his admission, the abscess was opened, and about three pints of fetid pus were evacuated from it. Next day he complained of dyspnœa and pain about the chest; on which account he was bled largely and freely purged. Two days afterwards, the symptoms having experienced little alleviation, he was again bled, and several leeches were applied to the chest, which was also extensively blistered, but without any mitigation of the pain, difficulty of breathing or the cough.

"On the 19th instant I visited the patient. The pulse was then very quick and feeble; the anxiety of the countenance was very distressing; the sweat hung upon his cheeks; he had occasional shiverings; he could not lie down, but was constantly in a sitting posture, rather reclining to the right side; the lips were rather purple; the wound on the back was healed. On striking the *right* side of the thorax with the hand a *dead sound* was heard, whereas a *hollow* sound was perceptible when the *left* side was struck. With the *stethoscope*, I could *not* detect *any respiratory murmur* on the *right* side, excepting immediately under the clavicle, where it was puerile. On the whole of the *left* side the *respiratory murmur* was *distinctly* heard, and was *louder* than it usually is in adults. I did not propose any remedies, thinking the operation of paracentesis the only chance; and that seemed so poor a one, that it was not pressed.

"On the 22nd, I again visited this patient, and found him apparently dying, with a pulse scarcely perceptible, a leaden countenance, and a glassy eye, with a general clammy sweat over the whole surface of the body. In addition to the indications before afforded by the stethoscope and percussion, we could now, by the Hippocratic succussion, distinctly hear a fluctuation in the chest." The *right* side of the chest was observed to be considerably larger than the *left;*—the *former*, measuring from sternum to spine, being 24 inches, whilst the latter measured only 22 inches. The man urgently desired some attempt to be made for his relief, and notwithstanding his almost dying state, it was determined that paracentesis of the thorax should be performed. Seven pints by measure of sero-purulent fluid were slowly evacuated by an opening made between the 5th and 6th ribs near their angles. At the commencement of the operation "there was positively no pulse at the wrist, but it could be felt near the axilla." He expressed himself much relieved during the operation, the breathing became more easy, and the pulse was again perceptible. He was ordered small quantities of brandy and gruel, and a plug of lint was introduced into the opening. On the 23d, he was in all respects better. A pint and a half of sero-purulent matter flowed from the opening when the plug was removed. "On the following day he was still doing well, and about the same quantity of fluid came from the opening as on the preceding day. The improvement, however, was not progressive. On the third day after the operation, the dyspnœa returned, and the pulse became quicker. He nevertheless survived to the 9th day; and on several occasions expressed much gratitude for the relief he had obtained by the operation."

*Dissection.* The *right lung* was completely collapsed to a size not much larger than a man's fist, and was situated in the upper part of the chest.* On cutting into its substance, it presented the appearance of spleen. The pleura pulmonalis was very much thickened, three or four times more so than is natural. The pleura costalis was also thickened, and there was a false membrane covering it of considerable thickness. A considerable quantity of sero-purulent fluid was found in the sac of the pleura. It was also evident that the abscess in the back was connected with the cavity of the thorax, as the remains of a communication were plainly traced. The *pericardium* and external surface of the heart bore evident marks of severe recent inflammation.

Dr. Hastings remarks, that the above case is "full of interest to the pathological investigator, whether we view it as an instance of certainty of the diagnosis to be obtained by the conjoined use of the stethoscope, percussion, and the Hippocratic succussion; or as pointing to the propriety under certain circumstances, of performing paracentesis when apparently scarcely any hope of life remains." The benefit derived from the operation was so great as to inspire hopes of recovery, and it is Dr. H's conviction, that, but for the supervention of the pericarditis, he would have recovered.

We perfectly concur in Dr. H's opinion, that, but for the supervention of the inflammation of the heart, the man would have recovered even under the circumstances under which the operation was performed; and much, very much is it to be regretted, that Dr. H. was not requested to use his stethoscope until a fortnight after the abscess was opened in the back. The pain, dyspnœa, &c. which followed the evacuation of the abscess, we apprehend arose from a fresh attack of pleuritic inflammation occasioned by the admission of air into the cavity of the thorax, with which the abscess communicated. The disease, instead of being imperfectly understood, would then have been thoroughly elucidated, and the preservation of the life of the patient, would in all probability have resulted from an earlier use of auscultation and percussion.

* Qy. was there any adhesion?

What the "evident marks of severe recent inflammation" upon the heart and pericardium were, Dr. H. does not inform us: we take it for granted, however, that there was a layer of coagulable lymph, and, most probably, there was also serous effusion into the pericardium. Supposing this to have been the case, a question occurs to us, when did the pericarditis come on? After a careful consideration of the case, we greatly suspect that it existed at Dr. H's first examination on the 19th: the state of the pulse—anxiety of countenance, and erect posture, belong much rather to the affection of the heart, than to the thoracic effusion. If our conjectures respecting the state of the heart be correct, it seems not improbable that the pericardium became inflamed along with the pleura after the evacuation of the abscess, on the fourth day after admission.

The indications furnished in this case, by the stethoscope and percussion were *only* those of effusion of *fluid* into the right cavity of the chest compressing the lung, the Hippocratic succussion shewed the presence of *air* also; but it is probable that the quantity was not great, and that, being situated in the top of the chest where the clavicle and pectoral muscle would prevent the results of percussion being very distinct, a more hollow sound would scarcely be elicited—or at least be readily overlooked.

*Case 3. Empyema from Chronic Pleurisy, opening on the anterior Part of the Chest; event favourable.* "On Feb. 26, 1825, John Bond, aged 25, a labouring servant, was received as an in-patient of the Worcester Infirmary, under my care. He was affected with difficulty of breathing, cough, and copious expectoration of a muco-purulent matter tinged with blood. There was considerable emaciation—pulse 100—tongue clean. He had been ill five weeks. The disease commenced with a pain in the left shoulder, and dyspnœa. About a fortnight afterwards, swelling made its appearance about the centre of the pectoral muscle on the left side; at that time, also, cough came on, attended with a very great expectoration of thick bloody matter. An incision was made into the swelling, and about a pint and a half of fluid was let out, after which the spitting began to abate. At the time of his admission into the Infirmary, there was a small opening about the centre of the pectoral muscle, from which a bloody purulent matter was discharged, but not in great quantity. There was emphysema of the cellular membrane for several inches surrounding the opening.

"On *percussion* of the *left* side of the chest, there was *very little sound* elicited in any part of it. The *right* side of the chest, however, gave a *hollow sound* when struck.

"With the *cylinder* a *respiratory murmur* was distinctly heard over the whole of the *right* side of the chest. On the *left* side of the chest, *no distinct respiratory murmur* was detected, but a *kind of humming noise* when the ear was applied to the cylinder: this was no where so conspicuous as on the left side of the back. If the cylinder was placed over the opening in the parietes of the thorax, when he coughed, a

*peculiar hissing noise* was heard, as if occasioned by the rushing of air through the opening."

Under Dr. Hastings' judicious management this man progressively improved, and was discharged from the Infirmary in good health, two months after his admission. The sound of the affected side gradually became more natural on percussion, and the respiration at the time of his discharge was nearly as distinct as on the right side.

The "kind of humming noise" heard by the cylinder in this case, we apprehend to have been what M. Collin designates *metallic respiration*—a phenomenon overlooked by Laennec. According to M. Collin, metallic respiration exists only in two morbid states, (1) where there is a fistulous communication between the cavity of the pleura and the bronchia, and (2) in a very large tuberculous excavation with compact adherent sides. In addition to these two states, we have met with a third, in which this phenomenon, as well as *metallic tinkling* occurs, viz. a communication with the cavity of the chest, by means of an external opening through the thoracic parietes, of which kind we feel pretty confident that Dr. Hastings' case was an example.

A fourth case of "empyema from chronic pleurisy, cured by paracentesis thoracis" was communicated by Mr. Stevenson, Jun. There appears to have been immense enlargement of the side and "an evident *fluctuation* between the ribs." Six pints and a half of fetid pus were evacuated by the operation—the case went on well, and the patient was able to enjoy the sports of the field a little more than two months afterwards.

Another case related by Dr. Hastings occurred thirteen years ago—a swelling appeared on the left side of the chest—four pints of sero-purulent fluid escaped from an opening made into the tumour, and the man recovered.

Dr. H. afterwards details two cases of *contraction of the chest* supposed to arise from chronic pleurisy. In one of them, which appears to have been of many years standing, "the 7th, 8th, and 9th ribs, on the left side, had fallen in considerably, and approximated together more than those on the opposite side. By percussion, there was no sound in this part of the left side of the chest, though there was in other parts of it. By the stethoscope, I discovered *pectoriloquism* near the angle of the ribs, in this contracted part of the chest, but not in other parts; the respiratory murmur being in all other parts natural."

With respect to the *pectoriloquism* in this case, Dr. H. thinks it may be presumed to arise from a cavity, formed, "either by an abscess bursting in the lungs, or by the softening of a tubercle." To us it appears much more probable, that instead of a cavity, there remains a *condensed state* of the pulmonary tissue which communicates the voice from the bronchial tubes: for there is no fact of which we are more certain, notwithstanding Laennec's observations to the contrary;* than that condensation of the substance of the lung, whether from hepatization,

* See "De l'Auscultation, t. i. p. 147-149.

or crude tubercles, does communicate resonance of the voice from the bronchial tubes situated in the condensed portion. We have also facts which prove that the same phenomena may be occasioned by œdema of the lungs, under some circumstances; and, from analogy, we conceive that the "apoplexie pulmonaire," when extensive, may be another cause.

We examined a few days ago a remarkable example of contraction of the chest—a patient of one of our medical friends, in which we found *resonance of the voice*, more or less approaching *pectoriloquism*, generally throughout the affected side. We attributed the phenomenon of the voice to an indurated state of the lung. In another case of an old man under our care, with *contracted chest*, and more or less *pectoriloquism* throughout the side, we apprehend there is general *dilatation of the bronchial tubes*.

We trust there are few of our readers who will not, after the perusal of the foregoing cases, regard the operation of empyema in a more just and favorable point of view than has hitherto been done; we also trust that they will stimulate a few to hasten to acquire that knowledge of auscultation and percussion, which will enable them to decide with more or less certainty, according to the proficiency they have made, whether there *is* or *is not* fluid in the cavity of the thorax. But whilst we recommend the operation to be more frequently resorted to, let us not be understood to advise it in cases of the *hydrothorax* of Cullen and other authors, for assuredly the man who should attempt it in such cases, would very rarely find it beneficial, and he would sometimes be disappointed in his search after fluid. The indications of auscultation and percussion, together with enlargement of the side, and bulging of the integuments between the ribs, are the only sure means of detecting the disease.

One of the most interesting cases in which the operation has been performed that has come to our knowledge, occurred in the practice of Mr. Jowett of Nottingham, and was read to the Medico-chirurgical Society upwards of twelve months since. The patient was a little girl in whom the disease had been quite insidious, and was only detected by an examination with the stethoscope, made with a forlorn hope of ascertaining a hopeless disease. Three pints of *pure pus* were evacuated and the patient recovered. During the progress of the cure a very extensive contraction of the chest took place, but one of the most remarkable and interesting features of the case was, that the thorax *afterwards enlarged again* so as nearly to recover its pristine dimensions,—a circumstance that had escaped the attention of previous observers. The case was accompanied with copious details of the general symptoms and the signs of auscultation and percussion.

Before closing this article, which has already exceeded the space we had allotted for it, we must endeavour to make room for the conclusions to which Dr. Hastings has come, and in almost all of which we entirely agree with this talented physician.

"1st, That a sero-purulent fluid may be collected, as the result of chronic inflammation, in each of the sacs of the pleura; but it is more commonly found in only one of them.

"2d, That the fluid may be only seated in a part of either of the right or left sac of the pleura, as in the superior or inferior part of one of these sacs. Thus a fluid may be found in the inferior part of the chest, resting on the diaphragm, and none in the superior part of the chest; the pleura pulmonalis and costalis, in the superior part of the chest, being united together by a false membrane.

"3d, That this inflammatory action, terminating in suppuration, may or may not be connected with disease in the cellular structure of the lungs; and, if it be connected, the disease, in the cellular structure, may be either of an inflammatory or tuberculous nature.

"4th, Softened tuberculous matter may find its way into the sac of the pleura, and become mixed with the sero-purulent effusion; and in this case a quantity of gas is also found in the sac of the pleura. But a gaseous fluid may be present in the sac of the pleura, combined with sero-purulent effusion, when the latter has solely arisen from inflammation of the pleura, unconnected with disease in the cellular texture of the lungs.

"5th, In sero-purulent effusion, the lung is always much compressed, and greatly diminished in size; and, in appearance, the lung often more resembles spleen than healthy lung.

"6th, That when there is a sero-purulent effusion into the cavity of the chest, unconnected with disorganization of the cellular tissue of the lungs, paracentesis thoracis speedily relieves the symptoms, and often effects a cure.

"7th, That, when a copious sero-purulent effusion into the cavity of the chest is connected with so much disease in the cellular structure of the lungs, as to forbid all hope of eventual recovery, paracentesis thoracis may frequently be performed, as a means of lessening the sufferings of the patient, and of prolonging his existence.

"8th, That the existence of adhesions, compressing and binding down the lung in chronic pleurisy, does not render the operation of paracentesis nugatory.

"9th, That the use of the stethoscope and percussion is of the highest importance in cases of empyema, as certainly pointing out the existence of a fluid in the chest, and the best situation in which the incision can be made, if the operation of paracentesis be performed.

"10th, That measurement of the two sides of the chest, will, in general, verify the indications thus obtained; the side which is affected with empyema being considerably larger than that which is free from effusion.

"11th, That the succussion of Hippocrates (de locis in homine, xxviii. p. 340), always affords evidence of fluctuation, when there is gas, as well as purulent matter, in the sac of the pleura.

"12th, That the fluid should not be rapidly discharged from the chest, and perhaps the operation would be more successful, if it were discharged in small quantities, at several separate times.

"13th, That it does not yet appear which is the most proper period of the effusion for performing the operation, whether early, before the effusion is very considerable, or late, when the fluid is in large quantity; but since our knowledge of this affection, by means of the stethoscope and percussion, will now become more precise, we may anticipate the speedy solution of this question by experience.

"14th, That active inflammation of the pleura does not often follow the operation: but this occurrence does occasionally take place.

"15th, That a spontaneous cure of effusion into the cavity of the chest in chronic pleurisy, not very uncommonly is brought about either by absorption of the fluid, or by the matter finding its way outwardly through the parietes of the chest; but it would not be right, on this account, to place our entire dependence on this salutary effort of nature.

"16th, That when persons recover from empyema, the side of the chest, which was the seat of the effusion, is often left in a contracted state; and this contraction, according to the extent of the previous effusion, may exist either in the whole or only in a part of the side of the chest that is affected.

"17th, That, in cases of empyema, under the most unfavourable appearances, we should not despond; for recovery is not impossible when the symptoms might lead us to pronounce the most gloomy prognosis." 36.

---

33. *Laryngeal Phthisis.* Two discoveries have recently been published in Germany—one by Soemerling, that herring-milt cures phthisis laryngea—while Dr. Fischer has discovered a "singular case" of the same disease which resisted the herring-milt!—It was not from want of perseverance that this poor man died, for he swallowed herring-milts daily for nearly a year. The larynx and trachea were found in a state of inflammation, and their lining membrane covered with a slimy purulent matter. The thyroid cartilage was as soft as cheese. The lungs were tuberculated. As there was no ulceration of the larynx we would have called this tracheal, rather than laryngeal phthisis. Be this as it may, we should have been more surprised if Dr. Fischer had discovered an exception to the failure, than to the success of the "celebrated herring-milt."

We should not have noticed the subject, had we not seen several instances where medical men deceived themselves by supposing they had cured laryngeal phthisis, when they had only cured chronic inflammation of the mucous membrane of the larynx and trachea. In this last disease, there will be cough, slow fever, muco-purulent expectoration, and emaciation—but still this is a very different thing from ulceration of the larynx, in which the cough is almost incessant—the discharge purulent and bloody—the voice at length lost—and the difficulty of swallowing often considerable. This is laryngeal phthisis—and who can cure *this* disease?

---

34. *Head-ache—Tic Douloureux—Biliary Disorder.** A young

* Dr. Granville. Med. and Phys. Journal, January.

lady, "moving in the first circles of society," led a rather too fashionable life during the *season* of 1824, and by the autumn made work for the doctors. She became affected with severe head-ache—her countenance being pallid—skin yellow—pupils natural—tongue clean—sleep tranquil—menstruation regular—bowels reported to be all right. The head-ache consisted in sharp, shooting pains, occupying for a short space of time, the left lateral and anterior portion of the head, "darting in and away," and occurring at very irregular periods of the day. On ordering evacuant medicines, the stools were found to be "large, figured, spiral, of a dark olive green, and exceedingly compact, shining, and offensive," and such had been their appearance for several months. Animal food was lessened—the farinaceous increased—an aperient pill, without mercury, was ordered at night, and a purgative draught. "At this period," says Dr. G. "there were none of the usual *organic* symptoms of *disturbed action* of the liver." Whatever Dr. G. may consider as *organic* symptoms, we should certainly have looked upon "dark olive green and offensive stools," as pretty indicative of *disturbed action* of the liver, whether there was pain or not in the region of that great laboratory. Under the above plan the head-ache was relieved, but the motions continued of the same description. She left town, but returned on the 20th November, greatly improved. In the course of a week, however, her countenance became very yellow—bowels inert—pulse quick. There was no head-ache, but soreness of the scalp covering the right fronto-parietal region. The purgative medicines were changed, and the action of the intestines invigorated. In the beginning of December the purgative medicines having been diminished, the shooting pains in the head returned, with torpid bowels, and discoloration of countenance. The original treatment was recommended, and another temporary improvement took place. On returning to London, in January, with a train of more formidable symptoms, which took place after waltzing somewhat immoderately at Bushy Palace, she had excruciating head-ache—vomiting of bile—foul tongue—costive bowels, &c. Here Dr. G. observes that he had ascertained, beyond a doubt, that mercurials produced an injurious influence on this young lady's nervous system (though there does not appear to have been any given during the preceding treatment) and, therefore, he only prescribed colocynth, rhubarb, and neutral salts. These not succeeding, he cautiously tried the blue pill, with considerable good effects on the bowels, but not without disturbance of the nervous system. The secretions now improved, and the head-ache diminished. Soon after this, the disease assumed another form. The pains of the head became irregularly periodical, coming on and going off suddenly, and often preceded by a sense of chilliness. "The pulse was, on all occasions, deep, slow, somewhat full, and irregular, with a rhythm which musicians call *stentato*." For the sake of his less-informed brethren we wish Dr. Granville had explained the nature of this musical pulse. The obstinacy of the head-ache now induced Dr. G. to order one general bleeding, which seemed to have the effect of removing, for a time, the pain in the head.

This amelioration did not last long, and we find her on the 20th January with sickness, head-ache, pain at the epigastrium, and foul tongue, &c. to which were now added, "exquisite soreness throughout the region of the liver." A consultation was requested, and Dr. Maton was proposed. An emetic, in the mean time, dislodged a large quantity of bilious secretions, sursum ac deorsum, and the patient was so much relieved, that Dr. Maton nearly lost a good job. On the 24th, however, we find Dr. M. in attendance. Rhubarb and hyoscyamus—salts and dill water. 29th, Another relapse of bilious disorder. Her countenance was now deeply yellow, with great head-ache, &c. Purgation and starvation again relieved the patient. On the 3d February, there was a new feature added to the Proteiform malady—pain in the left side of the face, neck, and external parts of the eye—which soon extended to the whole of the left side of the body, from the temple downwards—discharge of clear salt water from the stomach—bowels regular—pulse irregular and slow. The pil. sap. c. opio was prescribed, with camphor julep and liq. amm. acet. She was soon relieved. Dr. G. apologises for his minuteness of detail, by the wish to "shew the slow gradations by which it (the complaint) passed from costiveness to indigestion—from indigestion to head-ache—from head-ache to *rheumatic* affection of the muscles of the head, face, and neck—and from this to a more intense pain in the head, accompanied by weight, tinnitus aurium, deafness, shootings in the left eye, with a constant flow of tears, a full pulse, impossibility of laying the head on the pillow, total inability to bear light—all which latter aggregate symptoms had made their appearance insidiously, creeping on slowly, on the subsiding of the rheumatic pain in the face, at the time of last report." Some of Dr. Granville's readers may not be disposed to acquiesce in this pathological explanation, or concatenation. For our own parts we should be inclined to make shorter work of the explanation. The complaint, for all practical purposes, might be thus stated—great derangement of the digestive organs, followed by great derangement of the nervous system. We see little occasion for introducing the *rheumatic* link, and we very much doubt whether the pain was, at any time, really rheumatic.

The practice was now considerably altered. Whether the affection of the head arose or not from gastro-hepatic derangement, it was evident that the main attention must be directed to the former. Repeated local bleedings from the neighbourhood of the head gave repeated relief. A seton was also inserted in the nape of the neck—the bowels were kept open with neutral salts. After proceeding in this manner, for some time, Proteus again changed his shape. The head became free, and the stomach and liver bore the onus. The region of the latter organ became very painful. Having again employed—"for the 20th time" (this by no means appears on the record) "with no less injurious effects to the nerves, the pilula hydrargyri, and even the oxymuriate of mercury, we resolved on rubbing in the mercurial ointment on the region of the liver. This was done for ten or twelve days very freely, but no sensible effect was produced on the mucous membranes. The constitution seemed unsusceptible of the ordinary action of mercury." "One advantage, however, we derived from this course—namely, the removal of the enlargement and pain in the region of the liver." These symptoms disappearing, the head-ache again returned, with greater violence than ever. By purgatives they carried off prodigious quantities of black and green bile. This alternation in the seat of the disease, from the nervous to the chylopoietic system, and *vice versâ*, went on till the middle of April, by which time the physicians had before them "an extenuated patient, with a constitution broken down by unwearied sufferings, watchfulness, low diet, and fever—and still the call for purgatives and other lowering medicines continued imperative, for bad secretions still flowed, and the head-ache ceased not." It was about this time that Dr. Granville conceived, on minute examination, that the pain was of that kind which is termed neuralgic—in fact, that it had become *tic douloureux.* The carbonate of iron was now prescribed, and the patient was soon well in two or three days.

We have now endeavoured to condense into less than three pages, what has occupied eleven in the original. It is not our custom or our inclination to criticise the practice of our brethren; but every published case is open to comment, and one of the objects of the publisher ought always to be—the eliciting of remark. It is more easy, however, to give advice and pass opinions upon events, than to conduct them at the time. We give the practitioners credit, in this instance, for skill and attention; but, so far as we can judge by the evidence before us, we think most physicians will agree with us in believing that what was done at last, might have been done much sooner. We do not perceive that any bitters or tonics were prescribed in this young lady's case, at the time or after purgation was so freely used. Now, from some little attention to these complaints, we have long made it a rule, while the biliary derangement was so evident as in this case, to purge freely in the beginning with a combination of mercurial and other cathartics, regardless of the prejudices of the patient, and then to give bitter tonics, merely keeping up a moderate action on the bowels afterwards with aperients. We have daily opportunities of observing that continued purgation will produce continued bad or slimy secretions—and that by such course, the nervous system is greatly injured. We are convinced that the exhibition of bitter tonics, and especially the sulphate of quinine, during the administration of aperient and cathartic medicines, is almost a *sine quâ non*, in what are called derangements of the liver, stomach, and alimentary canal. To this may be added the immense advantage of country air and exercise in such cases.

---

35. *Hospital Reports.** M. Laennec's hospital report of La Charité for the second quarter of last year, does not contain matter of much interest. There are a few particulars, however, which are deserving of

* La Charité. Professeur Laennec, April to July, 1825.

notice. The mortality on the whole, was only one in ten. Of 20 cases of fever one proved fatal—and it was rather a remarkable case. The patient was a young man of 19, who came into the hospital on the 6th day of his illness, presenting very acute symptoms—great heat of surface, cephalalgia, abdominal pains, dysentery, redness of tongue, prostration of strength, pulmonary catarrh, &c. For these phenomena leeches were four times applied to the anus and abdomen—and rigid abstinence was enjoined. But the bowel complaint and abdominal pains continued—delirium supervened—the tongue became black—and he fell, in short, into a typhoid state, which went on till the 32d day, when sphacelus of the nates took place, followed by amelioration of the fever. The bowel-complaint, however, and the delirium still persisted. Bark and wine were now given, with opium. He rallied a good deal, but ultimately sunk on the 46th day of the fever. Here was a case for a Broussais or a Clutterbuck. On dissection, the contents of the head and thorax were found in a state of perfect integrity. The mucous membrane of the stomach was slightly tinged, while that of the intestines generally was very pale, with the exception of a small space near the termination of the ileum, where there were some small ulcerations, and marks of others which had healed. All the other viscera of the body were sound. M. Laennec justly remarks that here was a case of severe fever, where depletion was sparingly used (too sparingly in our opinion) at first, and where the *incendiary* treatment was employed during the last ten or twelve days of the disease—yet no man will pretend to say that the patient died of inflammation or its consequences, in any part of the body. The fact is, the patient was worn out by the long-continued orgasm of fever, not by the destruction of any organ in particular. He remembers when Brunonianism was at its height in France, and when the *incendiary* treatment was employed indiscriminately in all cases, and in all stages of fever; yet the ratio of mortality was little different from what it is at present, when leeches, starvation, and the antiphlogistic regimen are put in full requisition. An observation of the venerable Corvisart is here recorded, respecting the treatment of severe fevers—"we may treat them as we like, the result will be the same." "On peut faire ce que l'on voudra—cela ne se derange pas." We do not quite agree with Corvisart or Laennec upon this point—but we are quite satisfied that there is enough of foundation for their observation to destroy the doctrine, that fever is always dependent on inflammation of this or that organ in the body.

*Acute Rheumatism* continues to be treated by Laennec on the plan of the Italian physicians—namely, by large doses of tartarised antimony, from six to ten grains per diem. The success was not so great as formerly, in respect to the time of removing the disease; but the medicine produced no distressing effects in the large doses abovementioned.

A severe case of *phlebitis* occurred in a female, 27 years of age, and previously in good health. She entered the hospital on the 2d day of the complaint, presenting the following phenomena, for which no cause could be assigned. Face red—fever acute—pulse full, hard, and frequent—acute pain in the whole of the right arm, augmented by pressure, and exquisitely sensible on the inner edge of the biceps muscle, where the trunk of the bracheal vein could be felt, the size of the little finger, and as hard as a cord—erysipelatous redness in the course of the vein and its ramifications down to the hand—shining tumefaction of the member—tongue moist—no pain in the epigastrium—no disturbance of the digestive functions. The patient had not been bled, or wounded in any part of the arm. M. Laennec was afraid to bleed under these circumstances, and prescribed six grains of tartarised antimony, to be taken in divided doses, during the 24 hours. This was on the 6th August. On the 8th, (fourth day of the phlebitis) the fever was higher, the pulse harder, the pain and swelling of the arm increased. *Leeches to the anus.* The antimony was increased to nine grains, *per diem*, which produced some vomiting and purging. Some syrup of poppies was added and the medicine continued. 9th, The fever much diminished, with marked decrease of pain and swelling, excepting of the hand, which was of an enormous size. The antimony to be continued. 10th, The fever gone—the swelling scarcely perceptible—the vein could still be felt like a cord. From this time all the symptoms rapidly diminished, and she was discharged cured on the 24th August.

Laennec is afraid that some people may attribute the removal of the febrile symptoms, in this case, to the leeches applied to the anus, rather than to the full doses of antimony. His brethren on this side of the channel will not be of that opinion; and, considering the great danger, and even fatality, of phlebitis, the case here recorded is worthy of attention.

*Pulmonic Inflammation* is still treated on the Italian plan, with large doses of antimony, from six to ten or twelve grains *per diem*, but the results this quarter are not quite so satisfactory to us as to the parties concerned. Some of the cases, it must be confessed, were very unfavourable for any kind of treatment, such as complications with diseases of the heart, &c. therefore, we must yet suspend our judgment on this process. We cannot, however, but think that Laennec would succeed better if he combined the Italian with a little of the British practice.

*Affections of the Head* were treated by bleeding and antimony with success. One case of *chronic catarrh* is deserving of notice, as proving the confidence which may be placed in auscultation when practised by a trained ear. The patient was a coachman, 41 years of age, who had been subject to cough from his infancy, and was, in the course of the preceding few months, reduced to a state of complete marasmus. On examination with the stethoscope, M. Laennec pronounced the following diagnosis—"*considerable dilatation of the greater number of the bronchiæ, and disposition to gangrene of the lungs.*" This prediction was completely verified, but the case is to be detailed in the second edition of Laennec's work now in the press.

36. *Neuralgic Amaurosis.* [Dr. Belcher, Ed. Journ. Jan. 1826.] A man, aged 32, applied to Dr. Belcher on the 15th July, 1825, for total loss of vision of the left eye, the right being unimpaired. He was suffering acute pain of the temple, eye-ball, ear, cheek, and side of the head, the pain radiating along the branches of the pes anserinus, and aggravated by each attempt at mastication, or even by noise or a current of air. There was some degree of periodicity in the attacks. On further examination there were symptoms of well-marked amaurosis, as dilated immoveable pupil inobedient to the stimulus of light—the bottom of the eye of a black jet colour and glassy appearance. The superior palpebra was paralyzed and fallen down over the upper part of the eye-ball. He had been a fortnight deprived of vision—and twelve days before that, he had suffered from the neuralgic affection. In addition to these symptoms he had cephalalgia—morning pain in the stomach—bad taste in his mouth—furred tongue, and other marks of disorder of the digestive organs. The vascular system was unaffected, the mind despondent. The blue pill and aperients were ordered, and the vapour of liq. ammoniæ puræ, as recommended by Scarpa, was directed towards the eye, till lachrymation was induced. The bowels being well cleared, the carbonate of iron was given, in doses of 30 grains thrice a day, the alterative aperient being still continued. In four days the neuralgic paroxysms were mitigated, and some motion was observed in the upper eyelid. The amaurosis remained in statu quo. Next day he could discern the light, and move the eyelid freely. By the sixth day the neuralgic symptom had disappeared—the pupil contracts—the vision improving. The same treatment was regularly continued for about three weeks longer, at which time he was completely restored to vision and good digestion. He took, in all, 52 half-drachm doses of the carbonate of iron.

In this case (we agree with the very intelligent author) there is every reason to attribute the cure, or at all events the greater part of it, to the iron; though it would have been very injudicious not to have also attended to the state of the digestive organs. We have met with two cases, during the last year, of nearly a similar nature, where the disease readily gave way to alterative aperients and the sulphate of quinine, in conjunction with arsenic.

---

37. *Vesical Ulceration cured.* In a late number of the Edinburgh Medical Journal, Dr. Crowther relates a case, of which we shall here give the particulars. A young gentleman had been ill for some weeks, complaining of pain in the region of the bladder, augmented in micturition—quick pulse—irritative fever—emaciation. On examination, about three ounces of fetid pus were found daily in the urine. One day, on decanting the clear urine, Dr. C. discovered a number of transparent crystals mixed with the purulent discharge, from half to three eighths of an inch in length, with one end finely pointed like a lancet. He poured some boiling water on these and they soon dissolved.

Dr. C. ordered injections of *tepid water* to be thrown into the bladder twice a day, and the young gentleman was soon cured. Dr. C. makes sure that the ulcers "proceeded from wounds made in the coats of the bladder by the sharp spiculæ of the salt or gravel deposited from the urine." Now, did it not occur to the Doctor that these crystals were formed in the urine while cooling, after issuing from the bladder? This is our opinion of the case; for it is not very likely that Dr. C. threw in *tepid* injections of a higher temperature than 98°, at which temperature the urine stands in the bladder. Is it feasible that spiculæ or crystals would form in the bladder at this temperature?—We believe that Dr. C. cured his patient by the tepid injection; but not by preventing the formation of sharp spiculæ—it was rather by diluting the urine, too acrid by the solution of these salts, and too irritating to the coats of the bladder. Dr. C's facts may be correct, but we conceive his inferences or conclusions are erroneous.

---

### *Pharmacology.*

38. *Croton Tiglium.* The late Mr. Pope was a promising young chemist, but a premature death closed his prospects in this world. Shortly before his decease he presented a short paper to the Medico-Chirurgical Society, on a new preparation of the Croton Tiglium, of which we here give an analysis. It is well known that the common croton oil is a highly drastic purgative, hot and pungent on being swallowed, and producing great and painful irritation in the mouth and fauces for some hours afterwards. The uncertainty of its effects, Mr. Pope thought, arises from "an improper mode of preparing the oil," which has hitherto been by expression from the *entire* seeds. By numerous experiments Mr. Pope found that the acrid irritating property resided in the husk or shell of the seed, "while from the medulla alone a perfectly safe and efficacious purgative may be obtained."—The medulla appears to be the substance employed in India, as the ordinary purgative, by the natives, and for that purpose, is carefully separated from the epidermis, as well from the eye of the seed, in both of which the acrid principle intensely prevails. One seed made into a pill is sufficient for a dose. By subjecting the seeds to a similar manipulation, Mr. Pope procured the oil in question, which he submitted to the profession with some confidence. The dose is from one to two drops, either formed into pills, or diffused in any fluid by means of yolk of egg or mucilage. The best mode of exhibiting the medicine, however, is by dissolving it in a little alcohol, in the proportion of about one drop to half a drachm—in which state it may be more easily diffused in any simple fluid. By thus acting on an extended surface the purgative effect is more speedily insured. This alcoholic tincture has been used, we are informed, in various instances, with the best effects, particularly in children. Alcohol dissolves only about two thirds of the croton oil, but takes up the whole of the purgative principle. The acrid property of the epidermis is scarcely at all acted on by alcohol or ether. It gives out its acrimony readily to oil of turpentine, and also to olive oil. It produces intense burning heat in the tongue and fauces, which lasts for some hours, and raises pustules, if rubbed on the skin. It induced vomiting and purging when given to animals. The croton oil thus prepared has been used by many veterinary surgeons as the best purgative for horses. It acts without griping or irritation.

The oil, in its pure state, may now be had in great abundance from Mr. Pope's successor (Mr. Noakes, 96, Oxford-street), and also from Mr. Battley, of Fore-street, who prepares it himself. It is, comparatively speaking, very cheap. We believe the pure oil is not more than five shillings an ounce, whereas it was hitherto four times that sum.

---

39. *Chlorate of Soda.** This medicine, which is only to be used in solution, is prepared (by Garden, of Oxford-street, and Waugh, of Regent-street) by passing a stream of chlorine gas through a dilute solution of the subcarbonate of soda, in Woulfe's apparatus. When intended for inward use, care must be taken that there be little or no excess of chlorine in the solution. For external purposes, a slight excess will do no harm, but the contrary. The dose varies from one drachm to two ounces of the saturated solution to a glass of water thrice a day. For a lotion or gargle, Dr. Darling orders equal parts of water and the solution containing a slight excess of the chlorine.—This may be further diluted by the patient, if necessary.

This medicine was introduced to Dr. D.'s notice 18 years ago, by the late Dr. Scott of Bombay. Since our author's residence in London, he has been in the habit of giving it in chronic diseases of the skin, and in those bilious or rather dyspeptic disorders for which pure chlorine was recommended by Dr. S. Dr. D. soon found that it was a valuable medicine, and then began to try it externally, and has found it extremely useful in all those chronic affections of the skin for which stimulating lotions are usually recommended, " and in particular, that in cases of sloughing in the erythema, from local irritation, to which children are particularly liable, in impetigenous, and in prurigenous affections, it has proved successful in many instances, after the failure of all the remedies in common use." In affections of the mucous membranes (where they will admit of application) the remedy was still more effective, especially in those of the mouth, eyes and vagina. " It is strikingly beneficial in erythematous inflammation, and in ulcerations of the throat," when used as a gargle. Dr. D. has recently found that this medicine is effectual in controlling the inflammation of the mouth produced by mercury. It has, in his hands, arrested the salivation, when diligently applied at the commencement of ptyalism; and in the worst cases, when the flow of saliva is great, the ulceration extensive, and the pain severe, " it has given comparative ease in a few hours, checked the inflammatory action, and enabled the patient to take rest." " With this preparation at hand, salivation is no longer a dread to me; and consequently I can prescribe mercury with much less apprehension than formerly, whenever the free use of that remedy is desirable."—

* Dr. Darling. Med. Repos. Feb. 1826.

The inflammatory and irritative affections of the vagina have yielded, in Dr. D's practice, to this remedy, sooner than to any other. The case of a lady, who was afflicted with prurigo pudendi muliebris, is stated, when this solution gave almost instant relief even after every other application had failed. Not having any experience of the medicine ourselves, we can only lay before our readers the substance of Dr. Darling's paper.

